# Seven Billion Microcosms: Evolution within Human Microbiomes

**DOI:** 10.1128/mSystems.00171-17

**Published:** 2018-03-20

**Authors:** Tami D. Lieberman

**Affiliations:** a Institute for Medical Engineering and Sciences, Massachusetts Institute of Technology, Cambridge, Massachusetts, USA; b Department of Civil and Environmental Engineering, Massachusetts Institute of Technology, Cambridge, Massachusetts, USA

**Keywords:** evolution, human microbiome, microbial ecology, within-person evolution, evolutionary biology, genomics, intestinal colonization

## Abstract

Rational microbiome-based therapies may one day treat a wide range of diseases and promote wellness. Yet, we are still limited in our abilities to employ such therapies and to predict which bacterial strains have the potential to stably colonize a person. The Lieberman laboratory is working to close this knowledge gap and to develop an understanding of how individual species and strains behave in the human microbiome, including with regard to their niche ranges, survival strategies, and the degree to which they adapt to individual people. We employ system-level approaches, with a particular emphasis on using *de novo* mutations and evolutionary inference to reconstruct the history of bacterial lineages within individuals.

## Abstract (precis)

Rational microbiome-based therapies may one day treat a wide range of diseases and promote wellness. Yet, we are still limited in our abilities to employ such therapies and to predict which bacterial strains have the potential to stably colonize a person.

## PERSPECTIVE

Rational microbial therapeutics have the potential to treat a wide range of diseases and promote wellness. Fecal transplants have shown amazing efficacy in treating people with recurrent *Clostridium difficile* infections (1), and this may be just the beginning. Recurrent urinary tract infections have been treated with asymptomatic bacteria that outcompete pathogens (2). Even for diseases that are not caused by bacteria, microbiome-based therapies may one day improve drug metabolism, supply vital nutrients, and modulate the immune system.

However, we remain limited in our ability to precisely manipulate microbiomes. Much progress has been made in understanding our microbiomes, revealing that our gut microbiomes are relatively stable in composition and contain signatures of individuality (3, 4). Yet, we still cannot predict which strains will stably colonize an individual. While many strains introduced during fecal transplantation colonize people heavily treated with antibiotics, it is not clear how to stably add a new member to an established microbiome, particularly if a probiotic must compete with a closely related indigenous strain (5).

The Lieberman laboratory strives to close this knowledge gap and develop a deeper understanding of individual species and strains, including their colonization dynamics, challenges to survival *in vivo*, niche range, survival strategies, and within-person evolution. We employ evolutionary approaches to infer the dynamics of individual lineages in the complex reality of human-associated microbiomes and complement these *in vivo* retrospective studies with *in vitro* experiments. The majority of our current research is focused on species colonizing human skin and human digestive tracts.

## EVOLUTION IN THE MICROBIOME AND ITS IMPACT ON HUMAN HEALTH

A back-of-an-envelope calculation reveals that bacteria in your microbiome harbor an immense potential for within-person evolution. Each of the ~10^13^ bacteria in your body (6) has more than 10^6^ nucleotides of DNA. The error rate of DNA replication is greater than 10^−10^ mutations per nucleotide, and bacteria in the human body likely replicate on the order of once per day. Therefore, more than a billion point mutations were created within you today! Even if exceedingly few of these mutations confer a selective advantage, this still represents an enormous potential for adaptation within each of our bodies. Recombination and horizontal gene transfer further this potential.

During my postdoctoral work, my colleagues and I discovered that commensal bacteria do indeed rapidly adapt within us during healthy colonization (7). The discovery of within-person adaptation in the microbiome is exciting, as it pinpoints genes and pathways critical to bacterial survival *in vivo* and hints that bacteria in our microbiomes may need to adapt to particular environments within each of us.

Within-person evolution may in part explain the stability of the microbiome (3) and why probiotics so infrequently colonize healthy individuals (5); indigenous, preadapted microbiota may be better suited to the unique combination of selective forces within each individual. My lab is investigating the implications of within-person adaptation. On the host side, we are studying the degree to which within-person adaptation enables commensal bacteria to migrate to areas of disease (e.g., lesions of children with atopic dermatitis) and the feedback that this has on the human host. On the community side, we are investigating the associations between adaptive mutations and community resilience.

## RAPID EVOLUTION FOR STUDYING HUMAN MICROBIOMES

Evolution within the human body is not just an interesting phenomenon with potential implications, it is also a powerful tool to be leveraged for basic and translational discovery. I am optimistic that a focus on within-person evolution will gain rapid, mechanistic, and translatable insights into within-person ecosystems.

The particular ways that commensal bacteria diversify and adapt *in vivo* can give us much-needed insights into our understudied residents. The adaptive mutations that a strain acquires reveal its most pressing weaknesses and, in doing so, provide us with that strain’s perspective of its niche (8). Further, the genes and pathways mutated (or acquired via horizontal gene transfer) reveal the molecular strategies for surviving in the face of these selective forces. For example, our investigation of *Burkholderia dolosa* within-person evolution revealed an understudied oxygen-sensing pathway to be of critical importance (9) during long-term infections; further investigation demonstrated that this pathway regulates virulence traits in response to low oxygen (10).

Knowledge of survival strategies can be leveraged for the design of precise microbiome manipulations, particularly rationally designed probiotics. For example, we may find that resisting resident phage is essential for a particular species’ long-term colonization; such a discovery would suggest profiling an individual’s phage population to identify the bacterial strain most likely to survive in their microbiome. Alternatively, revealing an opportunistic pathogen’s weaknesses may inspire new antibacterial therapies. We therefore focus our studies on bacterial species and strains that are known to cause disease or are likely to have therapeutic benefits. We are studying, among other things, *Staphylococcus aureus* adaptation to atopic dermatitis lesions and *Fusobacterium nucleatum* adaptation to colon tumors. We perform high-throughput *in vitro* assays to assess the phenotypic consequences of identified mutations and collaborate to test their role in models of disease.

Bacterial evolution can also illuminate transmission, both within and between people. My lab seeks to understand when and from where we acquire our microbes. We use whole-genome phylogenetic analyses and simulations to model the spread of bacteria across people and across the body. We can thereby provide lower-bound estimates for the number of times that an individual or organ is colonized by distinct lineages (11) and identify potential sources of these lineages. We are currently combining this evolutionary approach with fine-grained sampling to understand how *Propionibacterium acnes* lineages colonize and spread on healthy skin, particularly across individual pores. I believe that culture-based approaches, when paired with clever experimental design, will be particularly useful for addressing questions of transmission; the time invested in colony isolation often reaps rewards in the form of clarity of results (more on this later).

## HUMAN MICROBIOMES FOR STUDYING RAPID ADAPTATION

Much of population genetic theory is predicated on the assumption that rapid adaptation is exceedingly rare. Under the standard model that we teach in classes and often hold in our minds, neutral drift and purifying selection dominate. Further, the standard model of adaptation considers a single mutation arising from a uniform background in which a new genotype competes only with its ancestral genotype. A large body of work has emerged over the past couple of decades demonstrating that rapid adaptation is common, as are “soft sweeps,” in which independent but similar adaptive mutations rise in frequency. Observations of rapid evolution and soft sweeps have been made for humans (12), other eukaryotes (13), bacteria in flasks (14), and bacteria within individual people (15). These observations have prompted calls for additional population genetic data that can aid in the development of a more complete theory. For example, it has been suggested that a better understanding of fluctuating selection might reconcile rapid adaptation with the signals of neutrality observed over longer time scales (13).

Human gut microbiomes provide an exciting opportunity to understand evolution in the real world. Billions of microcosms have already been established, with known ages and relationships to other microcosms and limited cross-microcosm immigration. These microcosms produce noninvasive community samples at regular intervals (stool) and can be studied intensively due to the low cost of sequencing. It is fairly straightforward to investigate the fate of adaptive mutations, their dynamics, and their genomic origins. While we cannot control nor measure everything about the biotic and abiotic conditions in these microcosms, we can still assess the dynamics and reproducibility of evolution, responses to perturbations (e.g., diet and fecal-microbiota transplants), and the interplay of rapid evolution and ecological dynamics. Data and theory connecting rapid evolution and ecology are sorely needed, as there is a growing appreciation that they act on similar time scales (16, 17).

## CULTURE-BASED APPROACHES ENABLE A TYPE OF “SINGLE-CELL” ANALYSIS

A favored approach in my laboratory is evolutionary inference from whole-genome sequences. Crucially, we do not depend on longitudinal sampling (though it is useful), because within-host evolution often leads to diversification that preserves a record of natural history within the host (15). We use this diversity in the same way that human evolutionary biologists compare extant primates, except that billions of within-person evolution experiments have already been performed.

While it is tempting to use metagenomic data to address questions about colonization and evolutionary dynamics, I often prefer culture-based sequencing. Culturing single colonies can be thought of as a type of single-cell analysis, enabling linkage inference for minority variants and the distinguishing of *de novo* mutations from preexisting variation, which cannot be done with metagenomics alone. Linkage information is essential for proper interpretation of evolutionary dynamics (7, 11). Current single-cell sequencing approaches result in incomplete coverage of the genome, have high error rates, and are technically challenging. We avoid these complications by using the bacteria themselves to replicate their genomes for us as they grow from single cells into colonies. We sequence hundreds to thousands of isolates per project, enabled by the low cost of isolate processing and sequencing (currently less than $25/isolate from colonies to a FASTQ file [18]).

We also employ, as needed, a variety of other approaches in the lab, including high-throughput microbiology, computational tool development and modeling, and interrogation of community spatial structure. When possible, we focus on the human environment, in order to rapidly translate discoveries of bacterial challenges to survival into prebiotic and antibiotic therapies.

## EXCITING TIME FOR STUDYING WITHIN-PERSON EVOLUTION IN THE MICROBIOME

The next 5 years will be an exciting time for human microbiome research, particularly for understanding within-person evolution and its connection to health and disease. As human trials begin to test the therapeutic efficacy of designed probiotics and other microbiome-targeted manipulations, a wealth of perturbation-associated data will become available for evolutionary and ecological study. Many groups have turned to culture-based approaches, and tens of thousands of reference genomes will soon become available from isolates cultured from healthy humans, covering nearly every major taxonomic group. Tens of thousands of metagenomes will soon be available. Many species and strains are being linked to both the nutrients on which they depend and the metabolites that they produce. These data sets will provide rich context for interpreting adaptive mutations found and other observations made within individual people and within disease cohorts. Integrating these diverse collections of data to make predictive models about how a microbiome will behave under a given perturbation will be challenging, and evolutionary and system-level perspectives will be key in tackling this challenge.
